# *frizzled 5* mutant zebrafish are genetically sensitised to developing microphthalmia and coloboma

**DOI:** 10.1242/dmm.052284

**Published:** 2025-06-10

**Authors:** Clinton Monfries, Stephen Carter, Paris Ataliotis, Aya Bseisu, Mahum Shaikh, Maria Hernández-Bejarano, Mohammed Fourteia, Mara Ioana Maftei, Rodrigo M. Young, Stephen W. Wilson, Gaia Gestri, Florencia Cavodeassi

**Affiliations:** ^1^ St George's School of Health and Medical Sciences, City St George's University of London, London SW17 0RE, UK; ^2^ Department of Cell and Developmental Biology, University College London, London WC1E 6BT, UK; ^3^ Centro de Biología Molecular Severo Ochoa, CSIC-UAM, Madrid, 28049, Spain; ^4^ UCL Institute of Ophthalmology, 11-43 Bath Street, London EC1V 9EL, UK; ^5^ Center for Integrative Biology, Universidad Mayor, 8580745 Huechuraba, Santiago, Chile

**Keywords:** Eye development, Microphthalmia, Coloboma, Wnt signalling, Zebrafish

## Abstract

Microphthalmia and coloboma are structural malformations of the eyes that arise from defective morphogenesis and are among the most severe defects associated with paediatric blindness. Frizzled class receptor 5 (*FZD5*) is a Wnt receptor expressed in the developing eye, and individuals with variants in *FZD5* exhibit microphthalmia/coloboma, supporting a role for this receptor in human eye formation. Here, we show that zebrafish *fzd5* mutants homozygous for complete loss-of-function or predicted dominant-negative alleles display no obvious eye defects during embryogenesis. Rather, they develop eye defects comparable to those described in humans only upon simultaneous abrogation of additional genes associated with ocular malformations. Thus, eye development can occur normally in the absence of Fzd5 in zebrafish, but mutants are sensitised to developing eye malformations. By exploiting the sensitised nature of the *fzd5* mutants, we further identified *angio-associated migratory cell protein* (*aamp*) as a novel gene involved in eye morphogenesis. Overall, our study confirms the importance of considering multiple genetic contributions when searching for the molecular aetiology of ocular malformations in humans.

## Teaser

**Summary:** This study provides insight into the mechanisms leading to eye malformation in *fzd5* mutants and highlights the importance of considering multiple genetic defects when searching for the molecular aetiology of ocular malformations in humans.

## INTRODUCTION

Microphthalmia, anophthalmia and coloboma (MAC) is a spectrum of ocular malformations arising from defects during early stages of eye formation that constitutes the most severe cause of childhood blindness (Plaisancié et al., 2019; Williamson and FitzPatrick, 2014; Yoon et al., 2020). Anophthalmia and microphthalmia describe, respectively, the absence of the eye or the presence of a reduced ocular globe, while coloboma typically refers to the persistence of an opening in the ventral portion of the eye. More than 100 MAC-associated genes have been identified in the past two decades. However, it is estimated that these genes explain only ∼30% of the cases described in human disease (Plaisancié et al., 2019; Williamson and FitzPatrick, 2014). One possible reason for this is that genetic variants may predispose to MAC, but defects are manifested only when combined with other risk factors or additional pathogenic gene variants. Therefore, such variants may potentially be overlooked by conventional searches, despite their relevance to understanding the aetiology of MAC.

Eye formation is critically dependent on the Wnt signalling pathway (Fuhrmann, 2008; Shah et al., 2023; van Amerongen and Nusse, 2009; Wiese et al., 2018). Wnt ligands bind to the extracellular domain of Frizzled (Fzd) receptors and a variety of co-receptors. Fzd receptors are seven-pass transmembrane proteins with an extracellular N-terminal domain that interacts with Wnt ligands. Upon Wnt/Fzd interaction, a conformational change promotes activation of Dishevelled (Dvl) (Bowin et al., 2023), inducing different responses in a highly context-dependent manner. Some of these involve transcriptional regulators, such as β-catenin, Yap or Jun, while others lead to modulation of cytoskeletal dynamics or calcium signalling (Shah et al., 2023; van Amerongen and Nusse, 2009).

Several of the components of the Wnt signalling network lead to eye malformations when mutated in animal models. For example, eye-specific disruption of β-catenin results in eye malformations in mouse (Hägglund et al., 2013; Westenskow et al., 2009), and functional abrogation of the Wnt co-receptor LDL-receptor-related protein 6 (LRP6) or the Wnt antagonist Dickkopf 1 (DKK1) result in microphthalmia and coloboma (Lieven and Rüther, 2011; Zhou et al., 2008). A role for the Wnt pathway in eye formation is further highlighted by the essential role of the Wnt/β-catenin pathway effector, *Tcf7l1*, during eye field specification (Kim et al., 2000; Andoniadou et al., 2011; Young et al., 2019), and by temporally dynamic expression of several Fzd genes in the eye primordium during embryogenesis (Fuhrmann et al., 2003; Nikaido et al., 2013). Among them, *fzd5* is the only Fzd receptor showing an eye-specific expression pattern (Cavodeassi et al., 2005; Fuhrmann et al., 2003; Sumanas and Ekker, 2001; Burns et al., 2008; Van Raay et al., 2005; Liu and Nathans, 2008; Nikaido et al., 2013).

Recent studies have uncovered a series of variants of *FZD5* associated with microphthalmia and/or coloboma in humans (Aubert-Mucca et al., 2021; Holt et al., 2022; Jiang et al., 2021; Liu et al., 2016). A total of 21 cases with missense, nonsense or frameshift variants have been described (Cortés-González et al., 2024; Holt et al., 2022). Only two variants have been functionally analysed *in vitro* and *in vivo* (Cortés-González et al., 2024; Liu et al., 2016). The first (Liu et al., 2016) is a frameshift variant leading to a premature termination codon, which generates a truncated form of the protein missing the transmembrane and the C-terminal domain. The truncated form of FZD5 was shown to work as a dominant negative, interfering with Wnt activity, and it has been proposed that this would explain the autosomal dominant inheritance pattern leading to ocular phenotypes described in these patients. The second (Cortés-González et al., 2024) is a missense variant leading to the replacement of a highly conserved proline by a leucine at the junction between the first intracellular domain and the second transmembrane domain (Pro267Leu). This variant has been shown to behave as a hypomorph, unable to efficiently transduce the Wnt signal (Cortés-González et al., 2024). In this case, the pattern of inheritance is recessive, and the variant in heterozygosis does not display any defective phenotype.

Conditional loss of function of *Fzd5* in the mouse eye leads to microphthalmia, coloboma and persistent foetal vasculature (Liu and Nathans, 2008), phenotypes that are exacerbated when the function of *Fzd8*, co-expressed with *Fzd5* in the eye primordium, is downregulated (Liu et al., 2012). *fzd5* anti-sense RNA approaches have also been described in *Xenopus* and zebrafish, which result in smaller eye primordia and reduced proliferation in the eye anlage (Cavodeassi et al., 2005; Liu et al., 2016; Van Raay et al., 2005).

Here, we describe the generation of four *fzd5* mutant alleles in the zebrafish, two reproducing a complete loss of function condition, and two reproducing a dominant-negative condition. Homozygote *fzd5* mutant embryos show no major ocular phenotype. However, quantification of eye size indicates that the eyes of *fzd5* homozygotes are smaller than those of heterozygotes or wild-type embryos. We show that further downregulating Wnt activity in these mutants exacerbates the ocular defects, suggesting that the mutants have compromised Wnt activity. In addition, interfering with the activity of other genes potentially involved in eye formation in these *fzd5* mutant lines results in eye malformations in an additive or synergistic way. Our results add novel insight into the mechanisms leading to eye phenotypes in *fzd5* mutants and highlight the importance of considering multiple genetic defects when searching for the molecular aetiology of ocular malformations in humans.

## RESULTS

### *fzd5* mutants undergo normal embryonic development and show subtle eye defects

**Fig. 1.**
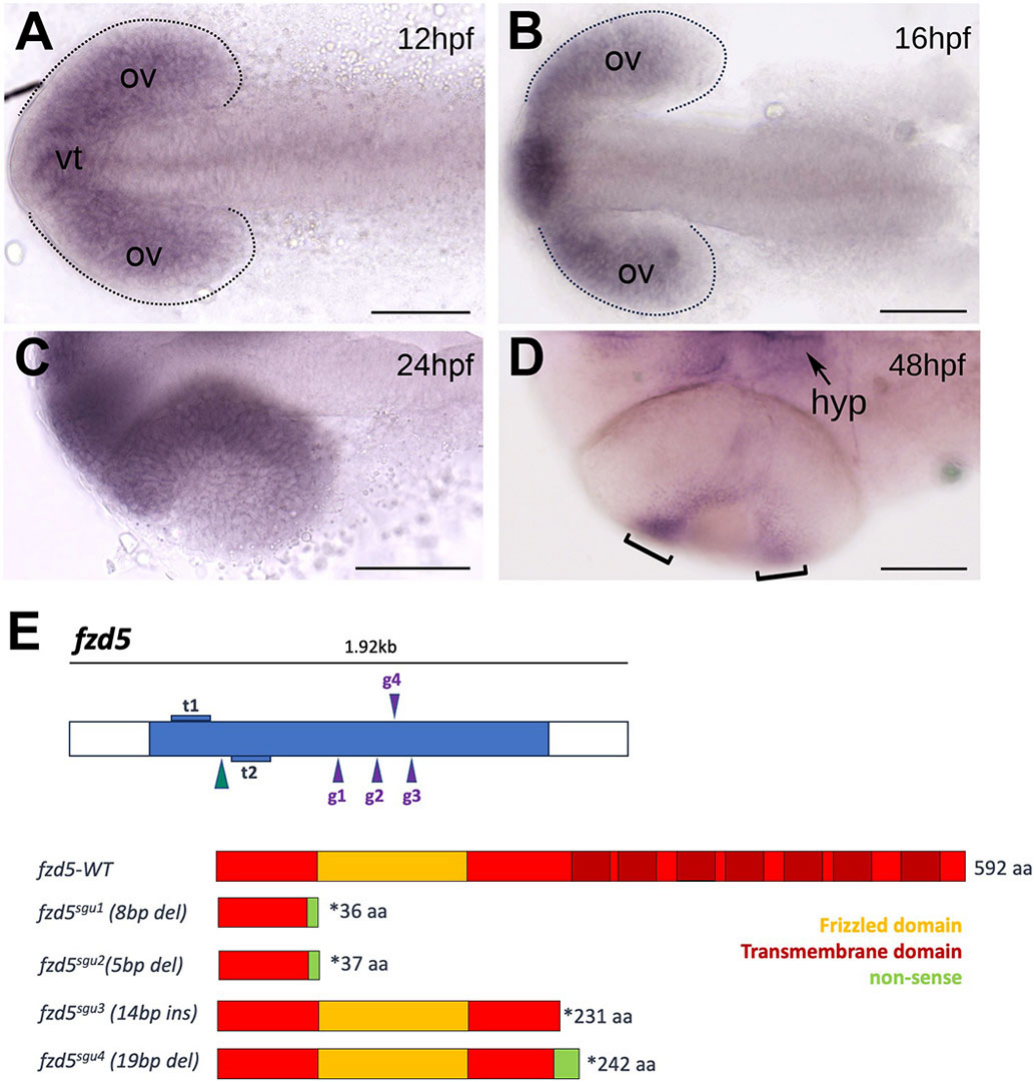
***fzd5* is expressed in the optic primordium throughout eye formation.** (A-D) Dorsal (A-C) or ventral (D) views, with anterior to the left, at the stage indicated in each panel. Dotted outlines in A and B highlight the optic vesicles. Brackets in D highlight the ciliary marginal zone (CMZ). (E) Schematic of the transcription activator-like effector nucleases (TALENs; t1 and t2) and guide RNAs (g1 to g4) used to generate the *fzd5* mutant alleles for this study, and their predicted protein products. aa, amino acids; hpf, h post-fertilisation; hyp, hypothalamus; ov, optic vesicles; vt, ventral telencephalon. Scale bars: 100 µm.

In zebrafish, *fzd5* expression starts at 10 h post-fertilisation (hpf) in the eye field (Cavodeassi et al., 2005) and continues at optic vesicle (Fig. 1A,B; 12-16 hpf) and optic cup stages (>24 hpf) up until the start of retinal differentiation (Fig. 1C). As the eye matures, *fzd5* expression is downregulated in the differentiated retina and only maintained in the ciliary marginal zone (CMZ) (Fig. 1D). Expression can also be detected throughout embryonic development in the ventral telencephalon and hypothalamus (Fig. 1A,D). To assess the requirement for Fzd5 in eye formation in zebrafish, we used genome editing to generate four mutant alleles (Fig. 1E): two predicted loss-of-function alleles (*sgu1* and *sgu2*) and two (*sgu3* and *sgu4*) mimicking the dominant-negative variant described in humans (Liu et al., 2016).

Two short deletions of eight (*fzd5^sgu1^*) or five (*fzd5*^*sgu2*^) nucleotides were generated by transcription activator-like effector nuclease (TALEN) injection (see Materials and Methods; Fig. 1E) and resulted in frameshifts and premature termination codons, giving rise to truncated products of 36 [p.(His18Leufs*37)] and 37 [p.(His18Leufs*38)] amino acids, respectively (Fig. 1E). These truncated forms lack an intact Wnt binding functional domain and are thus predicted to behave as complete loss-of-function mutations.

**Fig. 2.**
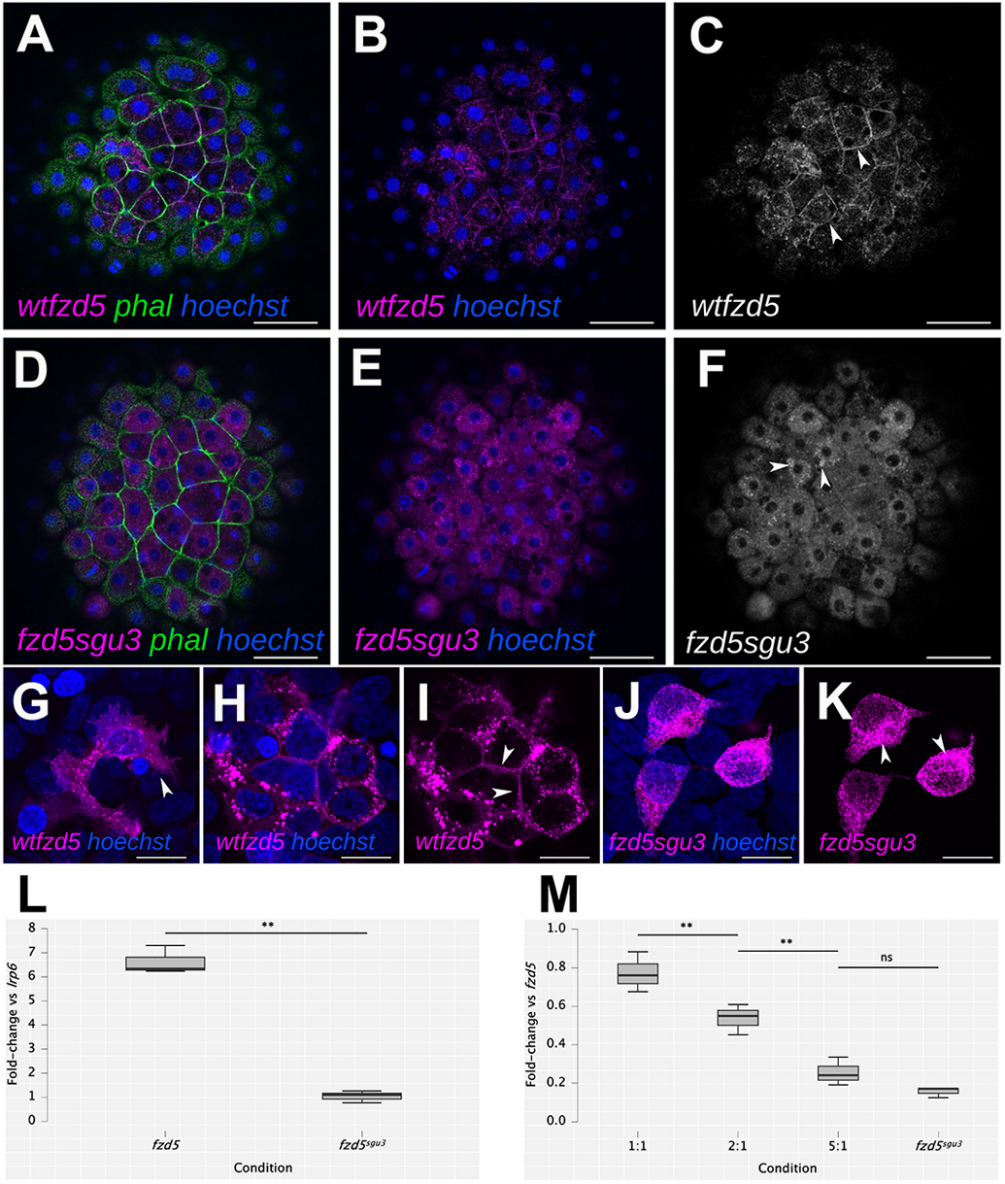
**The Fzd5^sgu3^ protein fails to localise to the cell membrane.** (A-K) Cell membrane localisation of wild-type Fzd5-RFP (*wt-fzd5*-RFP; magenta in A,B,G-I; grey in C; arrowheads in C,G,I) and punctate cytoplasmic accumulation of Fzd5^sgu3^-RFP (magenta in D,E,J,K; grey in F; arrowheads in F,K) in 4 hpf embryos injected with the corresponding mRNA (A-F), or in HEK293 cells transfected with the corresponding DNA construct (G-K). Embryos were counterstained with phalloidin-488 to reveal cell outlines (green) and Hoechst to reveal cell nuclei (blue). (L) Fold change in luciferase activity of HEK293 cells transfected with *lrp6+wt-fzd5-myc* and *lrp6+fzd5^sgu3^-myc* normalised to activity of *lrp6* alone. (M) Fold change in luciferase activity of co-transfections with *lrp6+wt-fzd5-myc* and increasing levels of *fzd5^sgu3^-myc* normalised to activity of *lrp6+wt-fzd5-myc* alone. Pairwise multiple Student’s *t*-test comparison between conditions in L and M reveal statistically significant changes in luciferase activity (L, *P*=0.002; M, *P*=0.007, *P*=0.003, *P*=0.091 from left to right). ns, not significant; **P<0.01. Data pooled from three experiments with four replicates each. Scale bars: 50 µm (A-F) or 10 µm (G-K).

The *fzd5^sgu3^* and *fzd5^sgu4^* alleles were generated by CRISPR-Cas9, injecting RNA guides designed to edit a region of zebrafish *fzd5* that is homologous to that affected in the dominant human *FZD5* variant described by Liu et al. (2016) (Fig. 1E). The *fzd5^sgu3^* allele harbours a 15-nucleotide insertion, generating a premature termination codon at position 231 [p.(Cys232*); Fig. 1E]. This form encodes an intact extracellular domain and no transmembrane domains (the first transmembrane domain starts at position 235 of the wild-type protein), and thus the truncated product of this allele is predicted to have dominant-negative effect. *fzd5^sgu4^* bears a 19-nucleotide deletion leading to a frameshift and a stretch of 13 non-sense amino acids before a premature termination codon at position 242 [p.(Ala228Argfs*243)]. The extracellular domain of *fzd5^sgu4^* is intact up to amino acid 228, and, similar to *fzd5^sgu3^*, no transmembrane domains are present (Fig. 1E). Consistent with a predicted truncation of Fzd5 produced by *fzd5^sgu3^*, a fusion form of *fzd5^sgu3^* tagged to red fluorescent protein (*fzd5^sgu3^*-RFP) fails to localise to the cell membrane (Fig. 2D-F,J,K, magenta or grey; arrowheads in Fig. 2F,K), in contrast to the cell-membrane localisation of a wild-type fusion form of *fzd5* (*wt-fzd5*-RFP) (Fig. 2A-C,G-I, magenta or grey; arrowheads in Fig. 2C,G,I).

A luciferase reporter assay (TOPFlash; Hua et al., 2018) in HEK293 cells revealed that the ability of *fzd5^sgu3^* to promote Wnt signalling was severely compromised (Fig. 2L). Cells transfected with 50 ng M50 8X TOPFlash only showed minimal luciferase activity. Robust activation of the Wnt pathway can be achieved by co-transfection of Fzd receptors with *lrp6* (Hua et al., 2018). Indeed, co-transfection of TOPFlash with wild-type *fzd5-myc* and *lrp6* resulted in a 6.6-fold increase in luciferase activity compared to co-transfection of TOPFlash with *lrp6* alone (Fig. 2L). However, co-transfection of *lrp6* with *fzd5^sgu3^-myc* (Fig. 2L) failed to activate above the levels seen with *lrp6* alone (Fig. 2L).

A dominant-negative effect of *fzd5^sgu3^* upon wild-type *fzd5*-induced TOPFlash activity was revealed by co-transfecting a constant level of wild-type *fzd5-myc* DNA with increasing amounts of *fzd5^sgu3^-myc* and normalising to luciferase activity for wild-type *fzd5-myc* alone (Fig. 2M). Equimolar amounts of *wt-fzd5* and *fzd5^sgu3^* resulted in ∼75% luciferase activity, a twofold excess of *fzd5^sgu3^* reduced this to 50% and a fivefold excess reduced this further to 25% (Fig. 2M). We then selected the *fzd5^sgu1^* and *fzd5^sgu3^* alleles for further analysis of the effect of loss-of-function and dominant-negative forms of Fzd5, respectively, on eye formation.

**Fig. 3.**
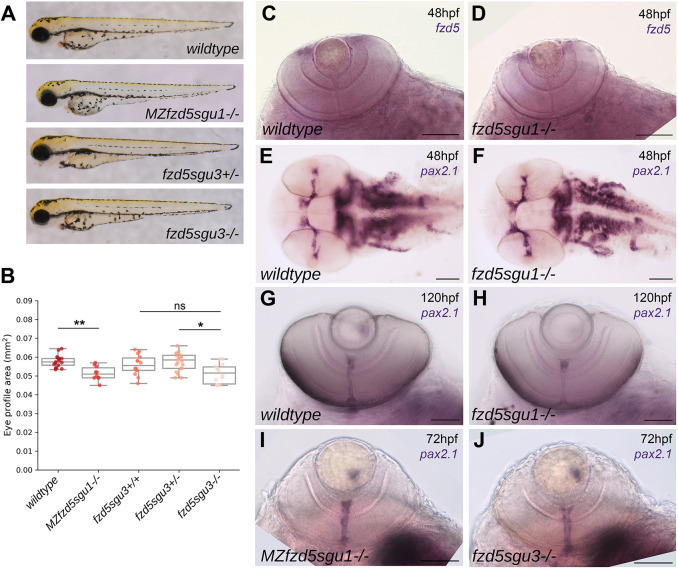
**Subtle eye defects in *fzd5^sgu1^*, *MZfzd5^sgu1^* and *fzd5^sgu3^* embryos.** (A) *MZfzd5^sgu1^* and *fzd5^sgu3^* 3 days post-fertilisation (dpf) larvae morphology compared to that of wild-type and heterozygote siblings. (B) Quantifications of projected eye area in the genotypic groups from A. Box plots represent the median and 25-75th percentiles; whiskers indicate the range of the data. Each data point represents one eye. A pairwise Tukey HSD post-hoc test revealed statistically significant eye size differences between the wild-type and the *MZfzd5^sgu1^* group (*P*=0.006748), and between the *fzd5^sgu3^* homozygote and heterozygote group (*P*=0.01591). ns, not significant; *P<0.05, **P<0.01. (C-J) Expression of *fzd5* in the CMZ (C,D) and *pax2.1* in the optic stalk/optic nerve (E-J) in *fzd5^sgu1^* (D,F,H), *MZfzd5^sgu1^* (I) and *fzd5^sgu3^* (J) embryos compared to wild-type controls (C,E,G). Embryo age is indicated in each panel. Scale bars: 100 µm.

No gross morphological defects were observed in homozygotes for both *fzd5^sgu1^* and *fzd5^sgu3^* alleles, but embryonic eyes were generally smaller. These differences, although subtle, were consistent and statistically significant between different genetic backgrounds when the projected eye area was measured (Fig. 3A,B and Fig. 7D). To determine whether there was a subtle/mild coloboma phenotype or a delay in choroid fissure fusion, we performed *in situ* hybridisation with *pax2.1*, a gene expressed in the lips of the choroid fissure and optic nerve, at the time of choroid fissure fusion. No difference in *pax2.1* expression was observed in either zygotic or maternal zygotic (MZ) *fzd5^sgu1^* mutants or zygotic *fzd5^sgu3^* mutants (Fig. 3E-J; an example of altered *pax2.1* expression associated to coloboma can be seen in Fig. 5F and Fig. S2B,C). No differences were observed in *fzd5* expression in the proliferating CMZ of zygotic *fzd5^sgu1^* mutants (Fig. 3C,D). Retinae of homozygous *fzd5^sgu1^* animals that survived to adulthood showed normal lamination and no obvious structural defects (Fig. S1).

### *fzd5* loss-of-function mutants are sensitised to expressing eye malformations

The findings that zygotic and *MZfzd5* homozygotes displayed only a subtle reduction in eye size during embryonic stages raised the possibility that other Fzds or Wnt pathway components compensate for the loss of Fzd5 to maintain pathway activity during eye development. For instance, several other Fzd genes are expressed throughout the anterior neural plate and eye primordia during embryogenesis (Nikaido et al., 2013). If so, we hypothesised that the mutants may be sensitised to the effect of other manipulations that disrupt Wnt signalling. To assess whether this was the case, we treated clutches of embryos derived from mating *fzd5^+/−^* parents with threshold levels of XAV-939, a selective antagonist of tankyrase activity that reduces Wnt/β-catenin activity (Kulak et al., 2015).

**Fig. 4.**
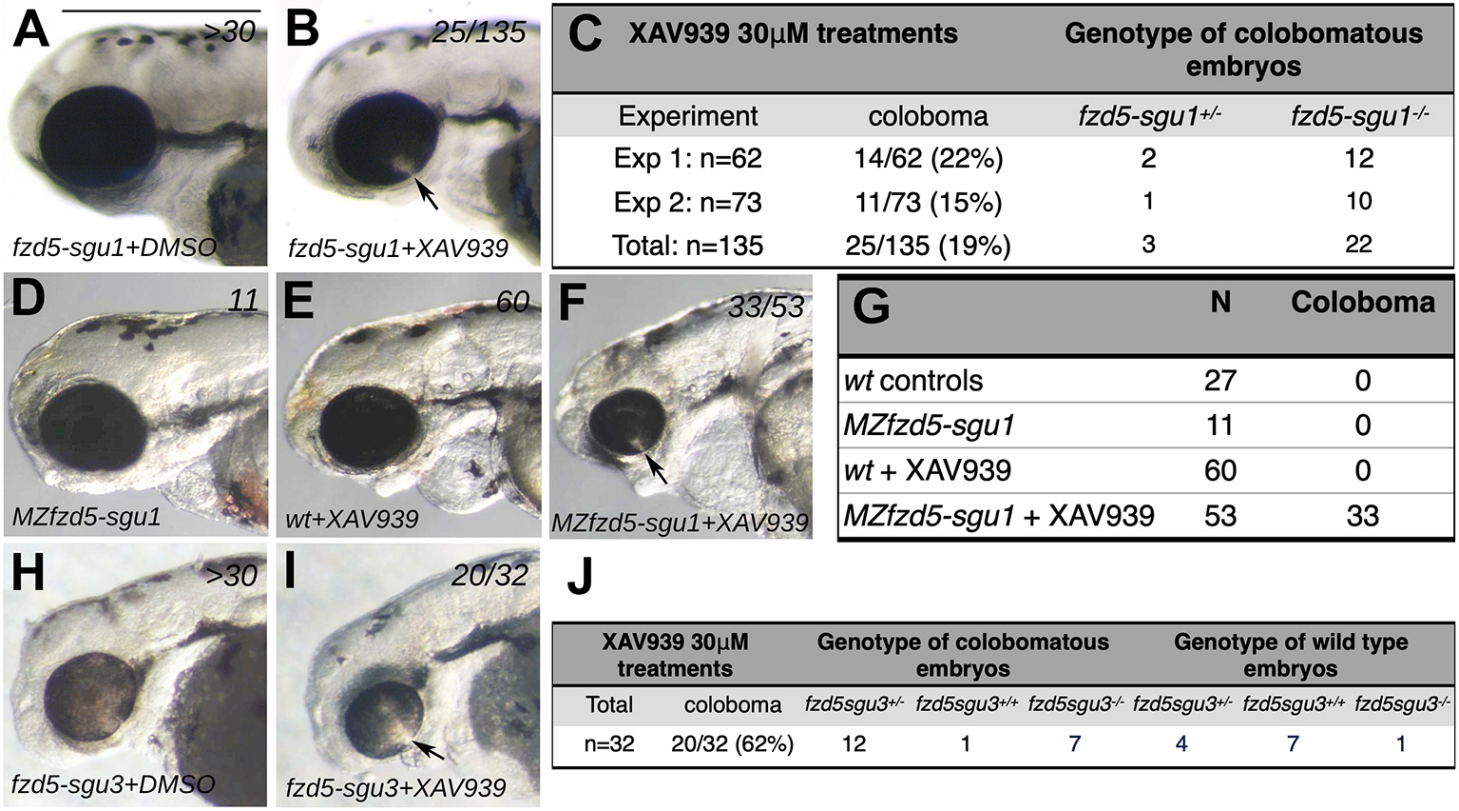
***fzd5^sgu1^* and *fzd5^sgu3^* are genetically sensitised to developing eye malformations.** (A,B,D-F,H,I) Lateral views, with anterior to the left, of 72 hpf embryos treated with DMSO (A,D,H) or XAV-939 (B,E,F,I). Colobomas (arrows in B,F,I) are evident in a subset of embryos derived from the incross of *fzd5^sgu1/+^* (B), *MZfzd5^sgu1^* (F) and *fzd5^sgu3/+^* XAV-939-treated embryos (I), a phenotype never observed in wild-type XAV-939-treated embryos (E). (C,G,J) Quantification of phenotypes observed, and their associated genotypes, in XAV-939-treated embryos derived from *fzd5^sgu1/+^* incross (C), wild-type or *MZfzd5^sgu1^* incross (G) and *fzd5^sgu3/+^* incross (J). Scale bar: 500 µm.

XAV-939 treatments of embryos derived from *fzd5^sgu1/+^* parents led to a subset of embryos displaying coloboma. From 135 treated embryos in two independent experiments, 25 (19%) showed coloboma (Fig. 4B, compare with DMSO-treated control in Fig. 4A). Subsequent genotyping confirmed that homozygote *fzd5^sgu1^* embryos were more sensitive to the treatment than wild types (Fig. 4A-C,E; Table S1). Indeed, 22/25 embryos in the coloboma group were *fzd5^sgu1^* homozygotes and none were wild type (Fig. 4C). These genotypic distributions (0 wild type: 3 heterozygote: 22 homozygote) markedly deviated from the typical Mendelian distributions of genotypes (1 wild type: 2 heterozygote: 1 homozygote; Table S1). No *fzd5^sgu1^* homozygotes were identified in a group of over 40 embryos without a coloboma phenotype (Fig. 4C; Table S1).

XAV-939 treatment on *MZfzd5^sgu1^* embryos resulted in 62% of them (33/53) showing coloboma (arrow in Fig. 4F, compare with DMSO-treated *MZfzd5^sgu1^* control embryos in Fig. 4D; see quantifications in Fig. 4G). A reduction in eye size also occurred in all *MZfzd5^sgu1^*-treated embryos. Notably, the coloboma/small eye phenotype observed in XAV-939-treated *MZfzd5^sgu1^* embryos was more severe than that observed in XAV-939-treated zygotic *fzd5^sgu1^* embryos (compare Fig. 4F and B). Consequently, although eye size phenotypes are similar in zygotic and *MZfzd5^sgu1^* embryos (Fig. 4A,B; see quantifications in Fig. 3A,B and Fig. 7D), the loss of maternal Fzd5 further compromises the ability of the forming eye to cope with additional modulations to Wnt pathway activity.

As for the *fzd5^sgu1^* allele, XAV-939 treatment of embryos derived from the mating of heterozygous *fzd5^sgu3^* parents led to a subset of embryos displaying coloboma (Fig. 4I, arrow; compare with DMSO-treated control embryo in Fig. 4H), but in this case the percentage of colobomatous embryos was much higher (20/32 total embryos, 62%; Fig. 4J) than that recovered from identical treatments in the *fzd5^sgu1^* background (19%; see Fig. 4C). A defective eye phenotype was recovered not only in homozygous embryos (7/8 embryos show coloboma) but also in most heterozygotes (12/16 heterozygotes show coloboma; Fig. 4J; Table S1). Thus, the distribution of genotypes in the coloboma group (1:12:7) and the group with wild-type eye morphology (7:4:1) markedly deviated from a Mendelian 1:2:1 distribution (Table S1). Collectively, these results suggest that *fzd5^sgu3/+^* heterozygote embryos are more sensitive to further abrogation of Wnt activity than *fzd5^sgu1/+^* embryos.

### Overexpression of a putative dominant-negative form of Fzd5 in the eye primordium leads to microphthalmia and coloboma

The experiments above suggest that a truncated form of Fzd5 expressed from its endogenous locus may act as a dominant-negative form to compromise the ability of the forming eye to maintain Wnt signalling. However, as this effect is only evident when the pathway is additionally compromised by drug treatment, perhaps the levels of the dominant-negative form present *in vivo* in the *fzd5^sgu3^* mutants do not significantly affect pathway activity on their own. To test this possibility, we used a transgenic line that expresses GAL4 under the control of the eye-specific *rx3* promoter (*tg{rx3:Gal4}*; Hernández-Bejarano et al., 2015; Weiss et al., 2012; Hernández-Bejarano et al., 2022) to express high levels of exogenous dominant-negative Fzd5 within the forming eye (UAS:*fzd5DN*; see Materials and Methods).

**Fig. 5.**
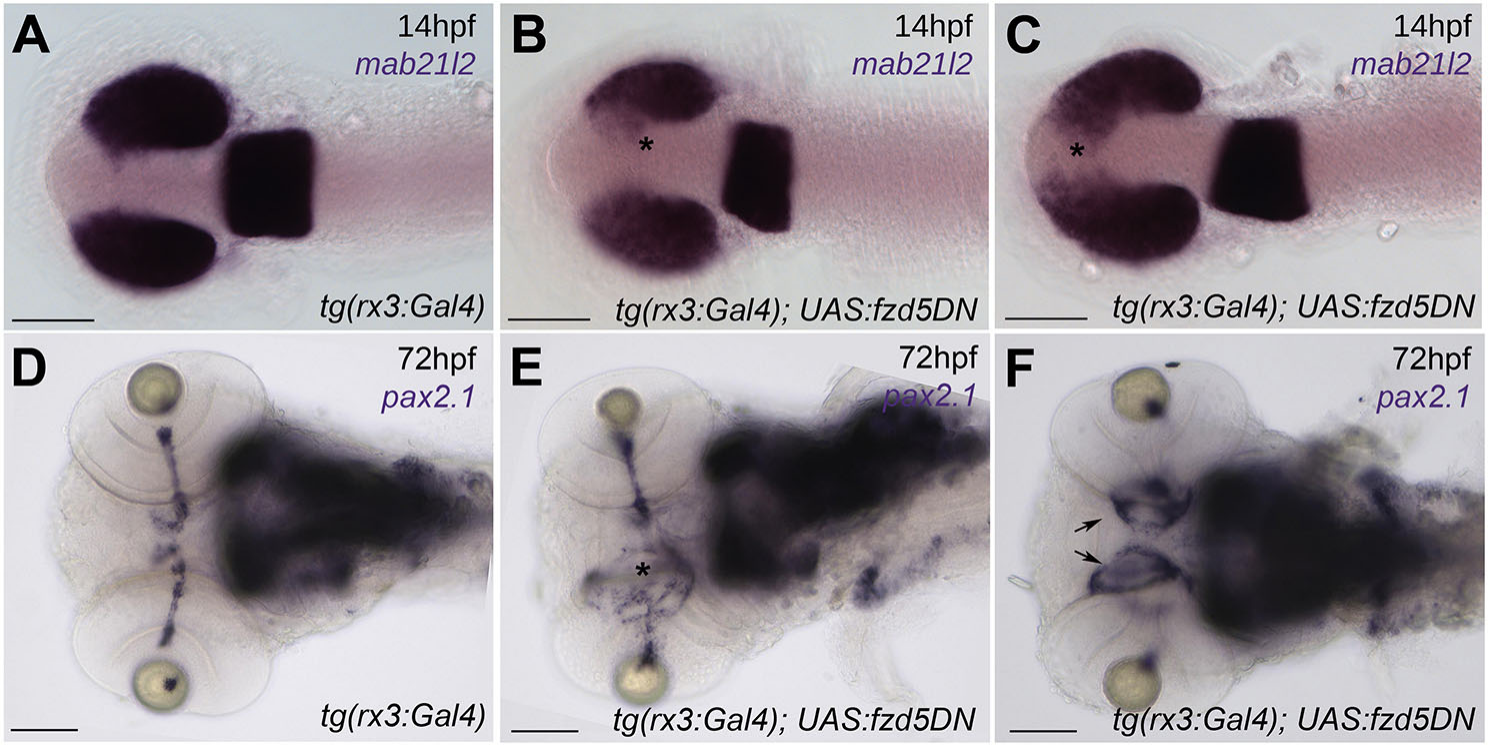
**Overexpression of a dominant-negative form of Fzd5 in the optic primordium results in eye defects.** (A-C) 14 hpf embryos stained with the pan retinal marker *mab21l2* showing incomplete optic vesicle evagination in *tg(rx3:Gal4);UAS:fzd5DN* [asterisks in B,C, compare with wild-type expression pattern in *tg(rx3:Gal4)* controls in A]. (D-F) *pax2.1* expression at 72 hpf highlighting optic nerve defects and optic disc coloboma in *tg(rx3:Gal4);UAS:fzd5DN* (E, asterisk; F, arrows) compared to *tg(rx3:Gal4)* controls (D). Panels show dorsal (A-C) or ventral (G-I) views of embryo heads with anterior to the left. Scale bars: 100 µm.

*tg{rx3:Gal4}*;UAS:*fzd5DN* embryos showed defective optic vesicle evagination resulting in optic disc and optic nerve defects. Control *tg{rx3:Gal4}* embryos expressed the pan retinal marker *mab21l2* in the optic vesicles, and no expression was observed in the forebrain midline (Fig. 5A). In contrast in *tg{rx3:Gal4}*;UAS:*fzd5DN* embryos, *mab21l2*-expressing cells were present in forebrain tissue between the eyes, suggesting incomplete optic vesicle evagination (Fig. 5B,C, asterisk; 11/11 embryos showed phenotype). By 72 hpf, optic disc coloboma was observed in a subset of the *tg{rx3:Gal4}*;UAS:*fzd5DN* embryos (Fig. 5E, asterisk; Fig. 5F, arrows; 7/17; compare with control in Fig. 5D).

These results suggest that interfering with Wnt/Fzd function in the retinal primordia leads to eyes with coloboma and optic nerve defects. In support of this conclusion, we observed optic nerve phenotypes upon overexpression of a dominant-negative form of the Wnt co-receptor Lrp6 [LRP6-DC, predicted to interfere with Wnt activity (Cavodeassi et al., 2005; Mao et al., 2001; Tamai et al., 2000)], either in the whole embryo (by *LRP6-DC* mRNA injection into one-cell-stage zebrafish embryos) or only in the eyes (by injection of a UAS:*LRP6-DC* construct into *tg{rx3:Gal4}* embryos; Fig. S2B,C,E, asterisk; compare with wild type in Fig. S2A,D).

### *fzd5* mutants are sensitised to revealing genetic interactions affecting eye formation

**Fig. 6.**
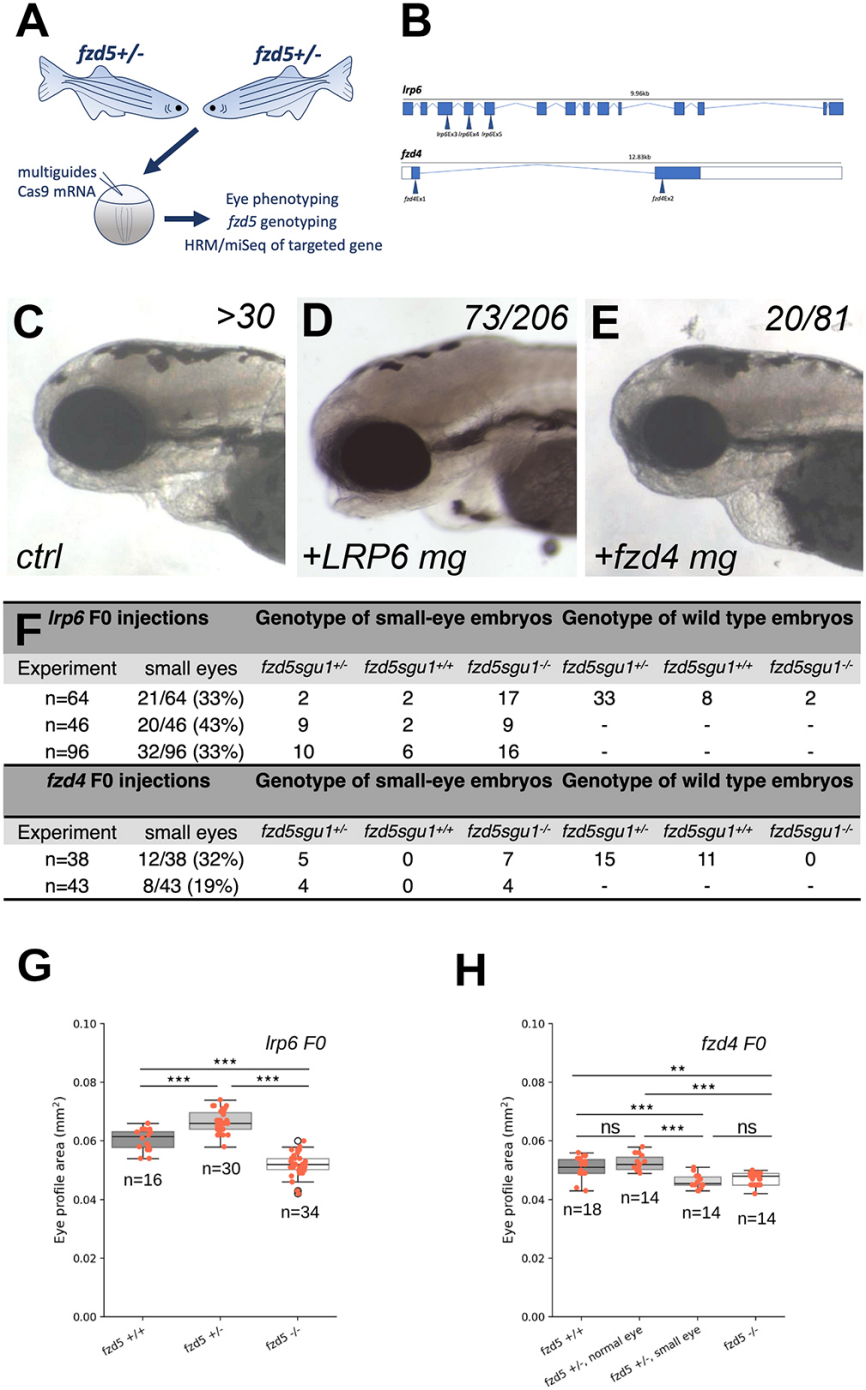
**Abrogation of Fzd4, but not Lrp6, function in *fzd5^sgu1^* heterozygote embryos results in smaller eyes.** (A) Experimental pipeline: *fzd5^sgu1/+^* adult carriers were mated, and offspring were injected at one-cell stage with CRISPR guides against the corresponding gene, plus Cas9 mRNA. Embryos were categorised according to eye phenotype, genotyped for *fzd5^sgu1^* mutation and sequenced to confirm the guides' gene editing. HRM, high-resolution melt. (B) Schematic representation of the guides used to interfere with *lrp6* (top) and *fzd4* (bottom) activity. (C-E) Lateral views, with anterior to the left, of 72 hpf control (C), *lrp6* (D) and *fzd4* F0 injected *fzd5^sgu1^* embryos. Numbers of embryos in the clutch displaying the phenotype are detailed at the top-right corner of each panel. (F) Quantification of *lrp6* and *fzd4* F0 injected embryos derived from a *fzd5^sgu1/+^* incross for three and two independent experiments, respectively, detailing the number of embryos showing small eyes and their associated genotypes. (G,H) Projected eye area quantifications of *fzd5^sgu1^* homozygotes and siblings F0 injected with *lrp6* (G) and *fzd4* (H) CRISPR guides. Box plots represent the median and 25-75th percentiles; whiskers indicate the range of the data. A pairwise Tukey HSD post-hoc test revealed statistically significant eye size differences between the different genotypic groups. ns, not significant; **P<0.01, ***P<0.001. Each data point represents one eye.

The results above show that further downregulating Wnt activity in homozygous *fzd5^−/−^* fish leads to exacerbated ocular phenotypes and suggest that these animals may be compromised in their ability to cope with additional genetic or environmental factors that challenge the robustness of Wnt signalling. To explore this in more detail, we next used a multiplex CRISPR-Cas9 approach to abrogate the function of *lrp6* and *fzd4* in *fzd5^sgu1^* mutant embryos (F0 approach; Fig. 6A,B; Table S1; Kroll et al., 2021). These two genes are expressed in the eyes, and while Lrp6 functions together with Fzd5 to activate the Wnt pathway, Fzd4 is a close paralogue of Fzd5 potentially showing functional redundancy with Fzd5.

**Fig. 7.**
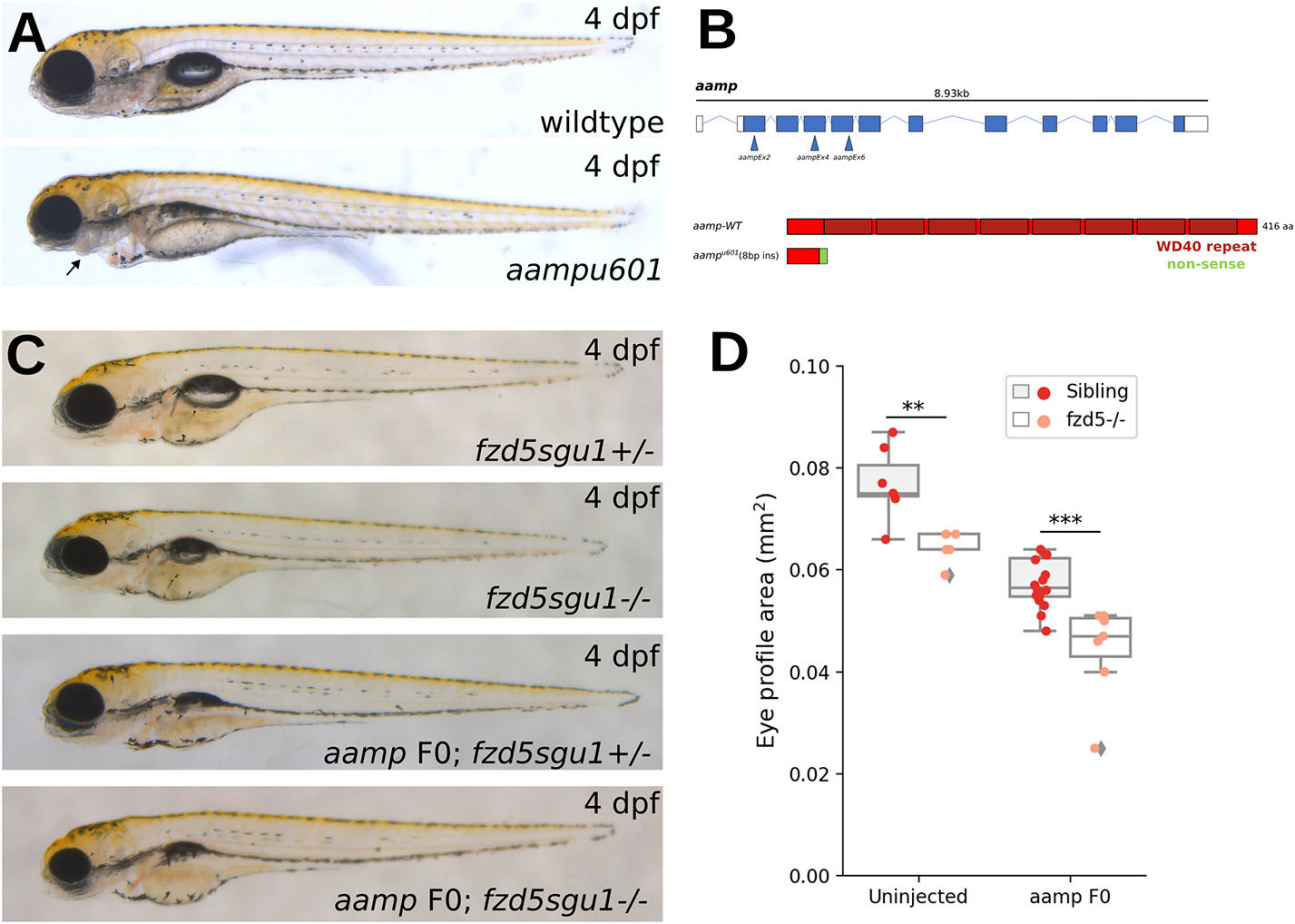
**More severe reduction in eye size in *fzd5^sgu1^* homozygotes devoid of *aamp* function.** (A) Phenotype of stable *aamp^u601^* mutant (bottom) 4 dpf larvae compared to wild type (top). Arrow indicates missing jaw cartilage. (B) Schematic of predicted protein product from *aamp^u601^* allele and representation of CRISPR guides used to target *aamp*. (C) Representative phenotypes of larvae derived from a *fzd5^sgu1/+^* incross uninjected or injected with *aamp* guides. Genotype status detailed at the bottom right of each panel. (D) Eye size quantifications of the four genotypic groups shown in C, showing additive effect of *fzd5* and *aamp* loss of function on eye size. Each data point represents one eye. A pairwise Tukey HSD post-hoc test revealed statistically significant eye size differences between the sibling and *fzd5^sgu1^* groups (**P=0.00765794), and between the *aampF0* sibling and *aampF0;fzd5^sgu1^* groups (***P=0.00033637).

Whereas abrogation of *lrp6* activity by F0 injection did not cause observable phenotypes in wild-type embryos (Fig. 6F), 33% of embryos (21/64) developing visibly smaller eyes were observed upon identical injections in embryos derived from *fzd5^sgu1/+^* parents (Fig. 6D, compare to wild type in Fig. 6C). Genotyping revealed that the small-eye group contained most of the homozygous *fzd5^sgu1^* embryos in the batch (17/19 homozygous embryos have small eyes; Fig. 6F), and the phenotypically wild-type group contained most of the heterozygotes and wild types (Fig. 6F). The genotypic distributions in both cases, as well as in two additional independent experiments, markedly deviate from typical Mendelian proportions (Fig. 6F; Table S1). Quantifying the projected eye area confirmed that the homozygous *fzd5^sgu1^* embryos had smaller eyes than the wild-type, phenotypically normal embryos (Fig. 6G; *fzd5^+/+^* versus *fzd5^−/−^*, *P*=1.10×10^−7^; *fzd5^+/−^* versus *fzd5 ^−/−^*, *P*=5.02×10^−22^). However, the percentage of eye area reduction between *lrp6* F0-injected *fzd5^sgu1^* homozygotes and wild types (∼14%) was not different from that seen in uninjected *fzd5^sgu1^* and *fzd5^sgu3^* homozygote embryos compared to wild types (see Figs 2 and 7). This result suggests that removing *lrp6* function does not exacerbate the small eye phenotype detected in *fzd5^sgu1^* homozygote animals. A lack of genetic interaction is also suggested by the observation that *lrp6* F0 injection did not lead to a reduction of projected eye area in *fzd5^sgu1^* heterozygotes (Fig. 6G).

*fzd4* abrogation by F0 injection led to small eyes in embryos derived from the mating of *fzd5^sgu1/+^* parents in two independent experiments [32% (12/38) or 19% (8/43) of small eyes, respectively; Fig. 6E]. Genotyping showed that the small-eye group contained all the *fzd5^sgu1^* homozygotes and a proportion of heterozygotes; no phenotype was observed abrogating *fzd4* in wild-type embryos (Fig. 6F; Table S1). Quantification of projected eye areas confirmed a statistically significant reduction in eye size in both *fzd5^sgu1^* homozygotes and small-eye *fzd5^sgu1/+^* heterozygotes compared to the normal-eye size groups (Fig. 6H; *fzd5^+/+^* versus *fzd5^−/−^*, *P*=0.005402; *fzd5^+/+^* versus *fzd5^+/−^*-small eyes, *P*=0.000992; *fzd5^+/−^*-normal eyes versus *fzd5^−/−^*, *P*=0.000015; *fzd5^+/−^*-normal eyes versus *fzd5^+/−^*-small eyes, *P*=0.000004). Thus, we concluded that embryos homozygous or heterozygous for the *fzd5^sgu1^* mutation were more prone than wild types to developing small eyes when *fzd4* activity was abrogated.

### Small-eye phenotypes are more severe in *fzd5; aamp* double mutants than in either single mutant

The genetic interactions presented above confirmed the sensitised nature of the *fzd5^sgu1^* genetic background and suggested that these mutants could be exploited to identify novel genes that, when disrupted, enhance susceptibility to eye malformations. To explore this, we selected six genes never associated with eye defects before, based on their co-expression with *fzd5* in the eye field as identified from single-cell RNA-sequencing datasets (Farrell et al., 2018). We then used the F0 approach to abrogate the function of these genes in fish heterozygous or homozygous for the *fzd5^sgu1^* allele. Only one of the six genes tested [*angio-associated migratory cell protein* (*aamp*)] showed a genetic interaction with the mutant *fzd5* allele. All the others [*starmaker* (*stm*), *Cbp/p300-interacting transactivator, with Glu/Asp-rich carboxy-terminal domain, 2* (*cited2*), *PPARG-related coactivator 1* (*pprc1*), *switching B cell complex subunit a/b* (*swap70a/b*)] did not show any phenotype in wild-type embryos and did not exacerbate the small-eye phenotype present in *fzd5* mutants (Fig. S3).

*aamp* encodes a poorly studied, but highly conserved, WD40 repeat domain-containing protein. *In vitro* studies suggest a role for *Aamp* in blood vessel development and smooth muscle migration, but no roles in eye formation have been reported (Beckner and Liotta, 1996; Vogt et al., 2008). *aamp* expression is highly enriched in the early optic vesicle and maintained in the eye primordium. As development progresses, its expression becomes restricted to the ciliary marginal zone, as well as other proliferative domains in the forebrain and tectum (Liu et al., 2016; Thisse and Thisse, 2004).

Abrogation of *aamp* generated by F0 injections resulted in smaller eyes at 4 days post-fertilisation (dpf), with an average reduction in projected eye area of 25% (Fig. 7A,C). The small-eye phenotype was reproduced and fully penetrant in a fish homozygous for a stable predicted loss-of-function allele (*aamp^u601^*) containing an 8 bp insertion in exon 2 (Fig. 7A,B; Fig. S4). In addition to small eyes, mutant *aamp^u601^*embryos showed a variable lower jaw reduction (Fig. 7A, arrow), and failed to inflate the swim bladder (Fig. 7A).

Abrogation of *aamp* function by F0 injection in embryos from a cross of *fzd5^sgu1/+^* parents resulted in an eye area reduction of 42% (Fig. 7D). This constituted a significant reduction in eye size compared to the 25% reduction in *aamp* F0 injections in a wild-type background and the 16% reduction observed in uninjected *fzd5^sgu1^* homozygote embryos (Fig. 7C,D). Thus, this approach uncovered a novel additive genetic interaction between loss of *aamp* and *fzd5* in the optic primordium.

## DISCUSSION

Variants of *FZD5* in humans have been shown to lead predominantly to isolated coloboma and less frequently to coloboma and microphthalmia (Holt et al., 2022; Jiang et al., 2021); but, to date, there is no clear understanding of the cause of this phenotypic variability. Here, we analysed two *fzd5* mutants in the zebrafish, one reproducing a complete loss-of-function condition (*fzd5^sgu1^*) and a second reproducing a predicted dominant-negative condition (*fzd5^sgu3^*). Eye formation seemed largely unaffected in homozygote embryos for both alleles, with only subtle reduction in eye size revealed upon quantification. However, coloboma was additionally present upon further downregulation of Wnt activity in the homozygote mutants.

Our results indicate that the *fzd5^sgu3^* allele compromises Wnt signalling more than *fzd5^sgu1^*. Downregulation of Wnt activity by XAV-939 treatment of embryos derived from a *fzd5^sgu3/+^* incross led to coloboma not only in *fzd5^sgu3^* homozygotes, but also in most of the heterozygote embryos. This is markedly different from the result obtained from XAV-939 treatments of embryos derived from a *fzd5^sgu1/+^* incross, in which coloboma was only observed in homozygote embryos. Collectively, our results suggest that zebrafish embryos can compensate for loss of Fzd5 activity, but *fzd5* mutants are predisposed to developing microphthalmia and coloboma in conditions in which Wnt activity is further compromised.

Our results do not entirely fit with the phenotypes described in humans. Human variants have been predominantly associated with isolated coloboma and, on rare occasions, to microphthalmia/coloboma, but only one case showing microphthalmia in the absence of coloboma has been reported (Holt et al., 2022; Jiang et al., 2021). Moreover, the mutants in zebrafish described in this study have a much milder phenotype than that observed in humans. Indeed, most of the human cases described in the literature show dominant inheritance of the mutant variants (Holt et al., 2022), and, to date, only one case has been described in humans carrying a variant with a recessive inheritance pattern (Cortés-González et al., 2024). Our interpretation is that the variants described in humans predominantly lead to more severe abrogation of Wnt activity than the conditions we described in this study. However, this does not explain why most of the cases described in humans display coloboma in the absence of microphthalmia.

The sensitised nature of the zebrafish *fzd5* mutants described in this study may provide an explanation for the dominant inheritance and variable expressivity of many of the alleles described in humans. Indeed, by simultaneously abrogating other genes potentially involved in eye formation in the *fzd5^sgu1^* background, we could identify novel genetic interactions on eye size with *fzd5* (*fzd4* and *aamp*). A proportion of the *FZD5* cases described in humans have been shown to carry variants also in other loci, and, at least in one case, compound variants of *FZD5* and another gene (*DDX3X*) required for eye morphogenesis and leading by itself to coloboma has been described (Holt et al., 2022). Thus, other patients may carry still unidentified variants of other genes contributing to the eye phenotypes described. Notably, most of the *FZD5* cases in the literature have been identified by whole-exome sequencing or by using customised next-generation sequencing panels of genes involved in ocular development, and thus potential variants in regulatory sequences of other interacting genes may have been overlooked. The identification of additional variants could be facilitated by assessing genetic interactions of candidate genes in the zebrafish mutants described in this study.

Although loss-of-function mutants for *lrp6* and *fzd4* in isolation have no effect on eye morphogenesis and eye size, *aamp* loss of function results in fully penetrant reduction of eye size. *Aamp* encodes a highly conserved WD40 repeat domain-containing protein (Beckner and Liotta, 1996; Vogt et al., 2008). In humans, WD40 repeat domains (WDRs) make up one of the most abundant protein–protein interaction domains, and WDR-containing proteins play important roles in nearly all major cellular signalling pathways (Haag et al., 2021). Variants of WDR proteins have been associated with various human pathologies including neurological disorders and holoprosencephaly (Haag et al., 2021), but the *in vivo* requirements for *aamp* are not known. Homozygous *aamp^U601^* mutants are not viable up to adulthood, suggesting that the mutant phenotype is not exclusive for the eye and corroborating *in vitro* studies that suggest a role for *aamp* in blood vessel development and smooth muscle migration (Beckner and Liotta, 1996; Vogt et al., 2008). Given the expression of *aamp* in proliferative niches of both the retina and the forebrain (Thisse and Thisse, 2004), we hypothesise that the small-eye phenotype is the result of a reduction in retina cell proliferation. Only seven *AAMP* loss-of-function cases were identified in the Genomics England 100,000 Genomes Project rare disease cohort. A subset of these patients show intellectual disabilities; however, there is no association with ocular defects, although subtle eye phenotypes may have been overlooked. Alternatively, eye phenotypes may only manifest in humans when *AAMP* is mutated in combination with other genes relevant for eye morphogenesis and associated with eye malformations, such as *FZD5*.

In summary, the animal models for *fzd5* presented in this study constitute valuable novel tools to unravel the genetic network cooperating with *fzd5* during eye formation, opening the exciting opportunity to exploit them to identify novel genetic interactions relevant to understand the aetiology of coloboma and microphthalmia.

## MATERIALS AND METHODS

### Fish lines and husbandry

AB and *tupl* wild-type zebrafish strains, the transgenic line *Tg{rx3::Gal4-VP16}^vu271Tg^* (Weiss et al., 2012), and mutant lines *fzd5^sgu1^*, *fzd5^sgu2^*, *fzd5^sgu3^*, *fzd5^sgu4^* and *aamp^U601^* were maintained and bred according to standard procedures (Aleström et al., 2020; Westerfield, 1993). Mutant lines were maintained in heterozygosis. With attentive husbandry, homozygous *fzd5^sgu1^* fish could be grown to adulthood and bred to obtain maternal zygotic mutant embryos. All experiments conform to the guidelines from the European Community Directive and the British (Animal Scientific Procedures Act 1986) legislation for the experimental use of animals.

### Generation of novel mutant lines

The *fzd5^sgu1^* and *fzd5^sgu2^* loss-of-function alleles were generated with the TALEN approach. TALEN arms were generated by Keith Joung's team and acquired from Addgene (TAL6232 and TAL3263; target sequence TCGGATTTTGGCTGCATGtcctgctgctgtttcaACTGTCTGGGCTCGGAGA; Sander et al., 2011). The TALEN arms target the 5′ region in the *fzd5* locus, between nucleotides 137 and 144 in the open reading frame. TALEN mRNAs were synthesised (mMessage mMachine SP6 kit, Ambion) and purified (NEB RNA cleanup kit) following manufacturer instructions. F0 founders were generated by co-injection of the two TALEN arm mRNAs into one-cell-stage AB/*tupl* embryos. Genotyping of F0 founders and their progeny was performed by CRISPR-STAT (Carrington et al., 2015) or high-resolution melt (HRM) analysis (Parant et al., 2009) from genomic DNA samples obtained from tail fin biopsies. The primers used are detailed in Table S2.

The target sequence to generate the *fzd5^sgu3^* and *fzd5^sgu4^* dominant-negative alleles was selected using the Ensembl Genome Browser. The optimal target DNA sequence on the *fzd5* open reading frame (ENSDARG00000025420) was identified following the recommendations from Hwang et al. (2013). To generate the guide RNAs, oligonucleotides (Table S2) were ordered from Integrated DNA Technologies (IDT), annealed and cloned in PDR274 or pCD039, linearised with DraI, transcribed *in vitro* (NEB Hiscribe RNA synthesis kit) and purified (NEB RNA cleanup kit) following manufacturer instructions. Cas9 mRNA was synthesised (mMessage mMachine SP6 kit, Ambion) and purified (NEB RNA cleanup kit) following manufacturer instructions. F0 founders were generated by co-injection of guide RNA (25 ng/µl) and Cas9 mRNA (300 ng/µl) into one-cell-stage AB/*tupl* embryos. Cleavage efficiency was assessed in pools of injected embryos by HRM analysis. Genotyping of F0 founders and their progeny was performed by HRM analysis from genomic DNA samples obtained from tail fin biopsies. The primers used are detailed in Table S2.

The *aamp^u601^* allele was generated by CRISPR/Cas9 (IDT) using a guide RNA targeting exon 2 of *aamp* (Table S2), just downstream of the start codon. The guide RNA was annealed to tracrRNA oligonucleotide (IDT #1072532), assembled with Cas9 protein (IDT #1081058) and injected into one-cell-stage AB/*tupl* embryos.

### Generation of F0 crispants

Guide RNAs to target *lrp6*, *fzd4*, *stm*, *cited2*, *pprc1*, *swap70a* and *swap70b* were generated as described above, and co-injected with Cas9 mRNA into AB/*tupl* embryos or embryos derived from the incross of *fzd5^sgu1^* heterozygous parents at one-cell stage. Guides were assessed for efficiency by genotyping injected wild-type embryos, and the minimum concentration of guides necessary to lead to efficient gene editing was subsequently selected to test genetic interactions. Best combinations often involved guides in adjacent exons to attempt to generate large deletions, in addition to small indels. Injected embryos did not show any non-specific phenotypes or developmental delay and were analysed to assess eye morphology, categorised according to phenotype and subsequently genotyped for *fzd5*. Identification of indels in *lrp6* and *fzd4*, and *fzd5* genotypic status was determined by HRM analysis on DNA samples obtained from individual embryonic tails. Identification of indels in *stm*, *cited2*, *pprc1*, *swap70a* and *swap70b* was determined by MiSeq analysis. At least two rounds of injection were done for each tested gene, and one of them was genotyped in full.

*aamp* loss of function was phenocopied by F0 injection as previously described (Kroll et al., 2021). Three synthetic RNA guides were designed (Table S2) and ordered from IDT. Guides were annealed to tracrRNA oligonucleotide (IDT #1072532), assembled with Cas9 protein (IDT #1081058) and injected into one-cell-stage wild-type embryos (Kroll et al., 2021). A subset of the injected embryos was genotyped by HRM analysis (primers described in Table S2) to confirm the presence of gene editing.

### Plasmid DNA constructs

*lrp6-DC*-pCS2+ was Addgene plasmid #27258; RRID: Addgene_27258). The *fzd5-DN* fragment was generated by amplifying the first 687 bp [encoding for the first 229 amino acids and lacking all transmembrane and cytoplasmic domains) from the *fzd5*-pCS2+ plasmid (Cavodeassi et al., 2005); primers detailed in Table S2]. The *lrp6-DC* and *fzd5-DN* fragments were subcloned into a UAS/Tol2 bidirectional plasmid (Distel et al., 2010; Hernández-Bejarano et al., 2015; Kajita et al., 2014).

pCS2-*wt-fzd5-RFP* (Cortés-González et al., 2024) was used as a template to generate *fzd5^sgu3^*-RFP, *fzd5*-myc and *fzd5^sgu3^*-myc fusion constructs using the NEB Q5 site-directed mutagenesis kit (E0554; primers detailed in Table S2).

### Microinjection and drug treatments

Suboptimal concentrations of XAV-939 (Sigma-Aldrich) were determined by treating wild-type embryos with a series between 100 µM and 15 µM. A concentration of 30 µM was selected for subsequent treatment. Embryos were collected and treated at shield stage. Eye phenotypes were scored at 3 dpf, and embryos were genotyped by HRM analysis as described above.

Overexpression of *fzd5-DN* and *lrp6-DC* in the optic vesicle was performed with the Gal4/UAS system (Halpern et al., 2008). 20-30 ng of *GFP:UAS:fzd5-DN* or *GFP:UAS:lrp6-DC* plasmid DNA was injected into the cell of one-cell-stage *Tg{rx3::Gal4-VP16}^vu271Tg^* (Weiss et al., 2012) embryos. Only embryos with homogeneous GFP expression in the optic vesicles were selected and processed for analysis (as described in Hernández-Bejarano et al., 2015). An average of 15 embryos were processed for each marker/stage analysed.

### Analysis of protein localisation

Fzd5 protein localisation was examined in zebrafish embryos using a *fzd5*-RFP C-terminal fusion (pCS2-*wt-fzd5-RFP*; Cortés-González et al., 2024). pCS2-*wt-fzd5-RFP* and pCS2-*fzd5^sgu3^-RFP* were used as templates to synthesise mRNA for injection (mMessage mMachine SP6 kit, Ambion). 200 pg of mRNA was injected into one-cell-stage fertilised embryos, allowed to develop at 30°C until dome stage (4.5 hpf) and fixed overnight in 4% paraformaldehyde. Embryos were briefly washed in PBS+0.3% Triton X-100 and incubated with phalloidin-FITC (at 0.5 µM, to detect subcortical actomyosin) and Hoechst (at 1 µg/ml, to detect DNA) in PBS+1% Triton X-100+1% DMSO for 4 h at room temperature, briefly washed in PBS and mounted in 1% low-melt-point agarose for imaging.

### Cell culture and cell transfections

HEK293 cells were maintained in Dulbecco's modified Eagle medium with 10% foetal calf serum at 37°C in a humidified atmosphere of 5% CO_2_. Transfections were carried out using polyethylenimine as described (Longo et al., 2013).

For immunofluorescence analysis, cells were plated on poly-L-lysine-coated glass coverslips and fixed in 4% paraformaldehyde at 24-48 h after transfection then stained with 10 µM Hoechst 33342. Coverslips were mounted on glass microscope slides using ProLong Diamond Antifade (Thermo Fisher Scientific).

Cells for TOPFlash assays were plated in quadruplicate on 24-well plates and transfected with M50 Super 8X TOPFlash (Addgene plasmid #12456; RRID: Addgene_12456) along with pRLTK (Promega) for normalisation. Activation of the Wnt pathway was achieved by co-transfection of *lrp6* and Fzd constructs, as described (Hua et al., 2018). Cells were processed 48 h after transfection with the Dual-Luciferase^®^ Reporter Assay System (Promega). Luminescence readings were made with a GloMax^®^ Discover Microplate Reader. Data were analysed by pairwise multiple Student’s *t*-test using Excel and JASP software (Version 0.17.3) on macOS 10.15.7.

### mRNA detection

Preparation of RNA antisense probes and whole-mount *in situ* hybridisation was performed as previously described (Hernández-Bejarano et al., 2015). *In situ* hybridised clutches derived from *fzd5^+/−^* incrosses were genotyped in full for *fzd5*, and all homozygote embryos were imaged and compared to wild-type controls.

### Sections of adult eyes and Haematoxylin and Eosin staining, and slidescanner operation

Adult tissue was collected and fixed as previously described (Moore et al., 2002), decalcified by incubation in 0.35 in EDTA pH 7.8, dehydrated in an ethanol series and cleared in methyl salicylate before embedding in wax. Sections were cut in a Leica RM2255 rotary microtome at 7 µm thickness. Sections were collected on slides coated with aminopropyl triethoxysilane, baked overnight at 37%, and stained with Haematoxylin and Eosin following standard protocols. High-resolution images of the slides were acquired on a Hamamatsu Nanozoomer 2.0-RS slidescanner, and images were selected and exported using NDP-View2 software.

### Eye size quantifications and statistical analysis

Embryos were staged according to the staging tables from Kimmel et al. (1995). All embryos looked morphologically similar, and no evidence of developmental delay was detected. Embryos were fixed in 4% paraformaldehyde and embedded in 3% methylcellulose for imaging. Images were acquired using a Nikon SMZ1270 microscope with a Plan Apo 1× WF WD 70 mm objective and a Leica MC190 HD camera, operated by Leica LAS EZ imaging software. Larvae were imaged lying on their sides such that only one eye was visible. Eye size measurement was performed in Fiji (Schindelin et al., 2012). Images were thresholded, a region of interest (ROI) was drawn to isolate the eye, and the thresholded area within the ROI was measured. Data were plotted using the python library Seaborn (Waskom, 2021), and statistical analysis was performed with the Pingouin package (Vallat, 2018).

### Imaging and data processing

*In situ* hybridised embryos and dissected eyes were mounted flat in a drop of glycerol, and dorsal or lateral images were acquired with a 20× (0.70 NA) dry lens using a Nikon Eclipse microscope connected to a digital camera (DS-Fi3) and operated by Nikon software (NIS-Elements).

Imaging of dome-stage embryos and transfected mammalian cells expressing wt-Fzd5-RFP and Fzd5^sgu3^-RFP fusions was performed in a Nikon A1R inverted confocal microscope with a 40× dry lens or a 100× oil immersion lens, respectively. Images were acquired with Nikon NIS Elements C software. Raw confocal images were processed with ImageJ.

Processed images were exported as TIFF files, and all figures were composed using Photoshop.

## Supplementary Material

10.1242/dmm.052284_sup1 Supplementary information
