# Supplementary material for: frizzled 5 mutant zebrafish are genetically sensitised to developing microphthalmia and coloboma
Source: Dis Model Mech. 2025 Jun 10;18(6):dmm052284. doi: 10.1242/dmm.052284 (PMC12182866; doi:10.1242/dmm.052284)
Supplement: Supplementary information [file dmm-18-052284-s1.pdf]

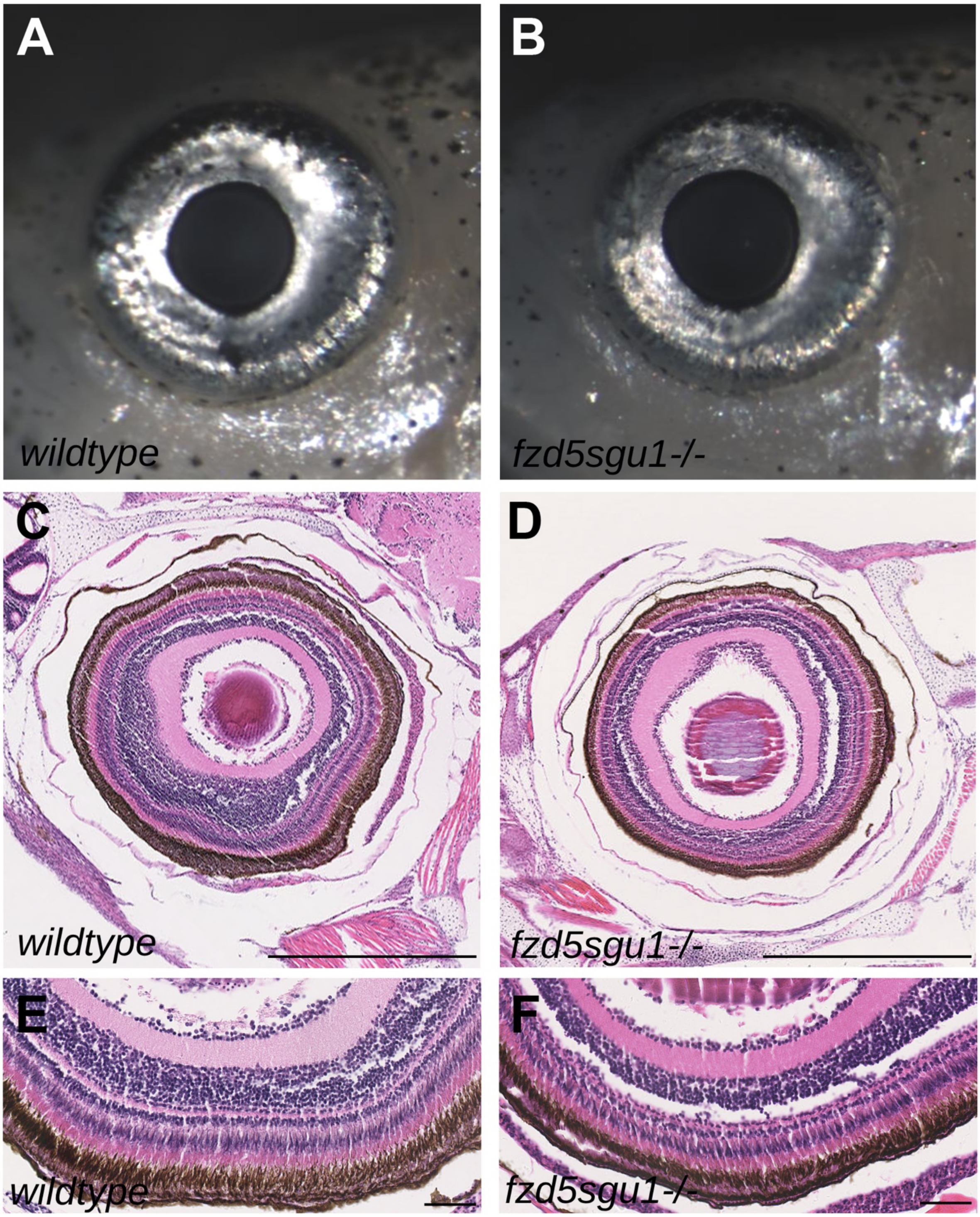

Fig. S1. Eye structure is not affected in *fzd5 ^{sgu1}* adult animals.

(A-B) lateral views with anterior to the left of 1 month-old *fzd5^{sgu1}* homozygote fish (B, compare with wild type in A).

(C-F) H&A sagittal sections through the eye of 1 month old *fzd5 ^{sgu1}* homozygote fish (D,F, compare with wildtype in C,E). Scale bar in C-D: 500µm; scale bar in E-F: 50 µm.

Disease Models & Mechanisms • Supplementary information

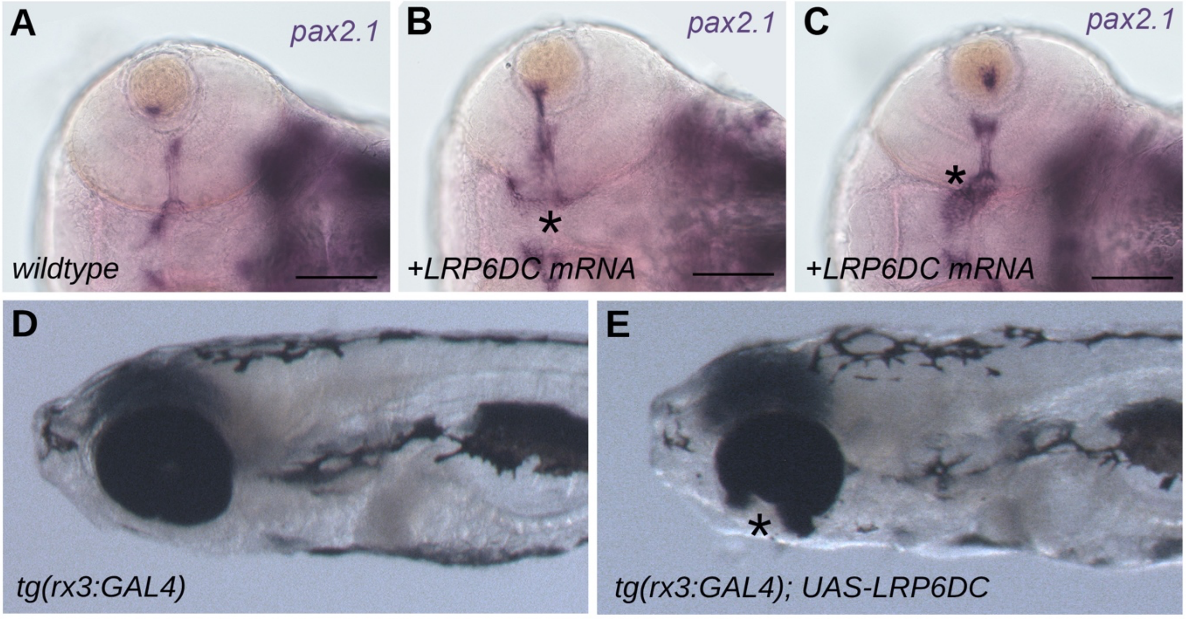

Fig. S2. Overexpression of *LRP6-DC* leads to coloboma and optic disc defects.
(A-C) *pax2.1* expression in the optic nerve at 72hpf highlighting choroid fissure closure/optic disc defects in *LRP6-DC* mRNA injected embryos (B-C, asterisks) as compared to wildtype (A).
(D-E) 5dpf larvae showing coloboma upon injection of *UAS-LRP6-DC* in the *tg(rx3:Gal4)* background (E) as compared to uninjected controls (D).
Images are dorsal (A-C) or lateral (D-E) views with anterior to the left. Scale bar: 100µm.

Disease Models & Mechanisms • Supplementary information

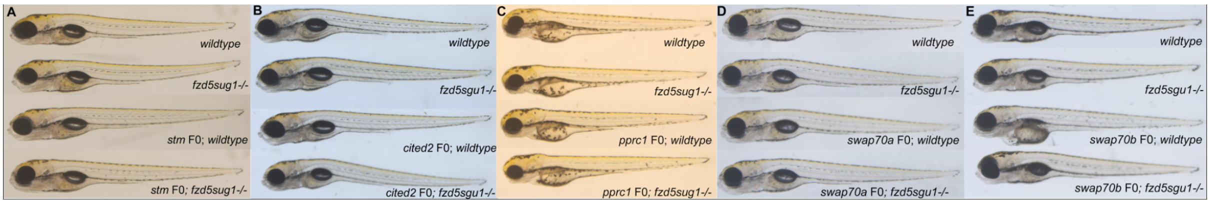

Fig. S3. F0 knockouts for *stm, cited2*, *pprc1*, *swap70a*, *swap70b* in wildtype and $fzd5^{sgu1}$ mutants show no overt phenotype.

(A-E) Representative phenotypes of larvae derived from a $fzd5^{sgu1/+}$ incross uninjected or injected with guide RNAs for: *stm* (A; n= 57/58 embryos show no differences from corresponding non injected genotypes); *cited2* (B; n=32/32 embryos show no differences from corresponding non injected genotypes); *pprc1* (C; n=55/58 embryos show no differences from corresponding non injected genotypes, among these 3/11 $fzd5^{sgu1}$ mutants have a more severe small eye phenotype as compared to non injected mutants) *swap70a* (D; 59/60 embryos show no differences from corresponding non injected genotypes); *swap70b* (E; 41/48 embryos show no differences from corresponding non injected genotypes; 7/48 embryos from different genotypes show heart oedema). Genotype status detailed at the bottom-right of each panel.

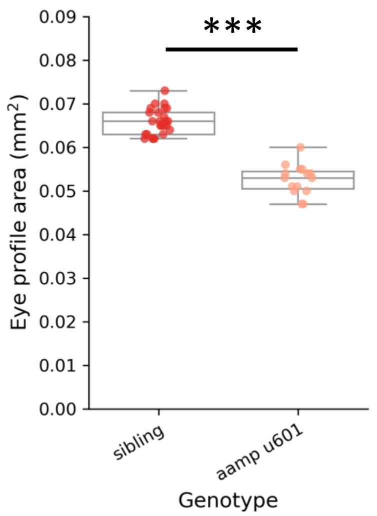

Fig. S4. *aamp^{u601}* homozygote embryos show significantly smaller eyes. Eye size quantifications showing a reduction of around 22% in eye size in the *aamp^{u601}* homozygotes as compared to their siblings. A pairwise Tukey HSD post-hoc test revealed statistically significant eye size differences between the sibling and the *aamp^{u601}* groups (p=1.613927e-12). Each data point represents one eye. 15 *aamp^{u601}* and 25 siblings from three independent clutches.

Table S1. Genotypic distributions (wildtype:heterozygote:homozygote) in the drug treatments and interaction conditions presented in this study.

Experiment	Genotypic distribution coloboma/ microphthalmia group	Genotypic distribution wild type group
fzd5-sgu1 + XAV-939	0:3:22	10:22:0
fzd5-sgu3 + XAV-939	1:12:7	7:4:1
fzd5-sgu1 + *LRP6* multiguides exp1	2:2:17	8:33:2
fzd5-sgu1 + *LRP6* multiguides exp2	2:9:9	-
fzd5-sgu1 + *LRP6* multiguides exp3	6:10:16	-
fzd5-sgu1 + *fzd4* multiguides exp1	0:5:7	11:15:0
fzd5-sgu1 + *fzd4* multiguides exp2	0:4:4	-

Table S2. Primers and guides used in this study.

Primer/Crispr guide	Sequence
**Genotyping *fzd5-LOF* sgu1 and sgu2 alleles**	
fzd5-LOF alleles HRM fwd	TTTCACCATGGAGACCTCAG
fzd5-LOF alleles HRM rev	CAGTGATGGGCTCACACAC
**Generation and genotyping *fzd5-DN* sgu3 and sgu4 alleles**	
fzd5-DN SP6 F2 Crispr guide	TAGAAAAGTAGGGTTGGTGGCA
fzd5-DN T7 F3 Crispr guide	TAGGTGAAGGTGCGCTCATCCT
fzd5-DN SP6 F4 Crispr guide	TAGAGCGCACCTTCACCACCTTC
fzd5-DN SP6 F5 Crispr guide	TAGATGAGGAAAGTGGCCACCG
fzd5-DN alleles HRM fwd	GCACATCCTCTTCATAACCGT
fzd5-DN alleles HRM rev	GACACAAAGCAAAGCACCGAC
***lrp6* guides and HRM genotyping primers**	
lrp6 -Ex3-T7-F Crispr guide	TAGGAGCCGTCCAGGTTGGAG
lrp6 -Ex3-F2 HRM fwd	CAGCAGCTCTACTGGGCC
lrp6 -INT3-R2 HRM rev	CACGTGCGCTGGTGACGGAG
lrp6 -Ex4-SP6-F1 Crispr guide	TAGAGGACACACTCTTCTGGA
lrp6 -Ex4-F1 HRM fwd	GGAGGTGGTGGTGAAGGGC
lrp6 -Ex4-R3 HRM rev	GTTTGTGGCACGAGTGAATG
lrp6-Ex5-T7-F1 Crispr guide	TAGGCGAGCCCCTGCTCTGTG
lrp6-INT5-F1 HRM fwd	CTTCATCAACGTGTCTTCTCATGT
lrp6-Ex5-R2 HRM rev	CAGCAAACACAGATGAGAACATC
***lrp6* NGS primers**	
lrp6 -Ex3-Mi-F	TCGTCGGCAGCGTCAGATGTGTATAAGAGACAG GGTTCATGTACTGGACGGACTGG
lrp6 -Ex3-Mi-R	GTCTCGTGGGCTCGGAGATGTGTATAAGAGACAG CACGTGCGCTGGTGACGGAGGTGTTC
lrp6 -Ex4-Mi-F	TCGTCGGCAGCGTCAGATGTGTATAAGAGACAG GGAGGTGGTGGTGAAGGGCTCTCTG
lrp6 -Ex4-Mi-R	GTCTCGTGGGCTCGGAGATGTGTATAAGAGACAG CATGGGCTGGCGCTGCTGGCTG
***fz4* guides and HRM genotyping primers**	
fzd4 -Ex1-SP6-F5 Crispr guide	TAGACCAAGATGCCGAATCTGG
fzd4 -F2 HRM fwd	GTTCGGGGACGAGGAGGA
fzd4 -Ex1-R2 HRM rev	GTGTGTAGGGTCTGGTTACAGTG
fzd4 -Ex2-T7-F1 Crispr guide	TAGGCACCTTCTCCGTGCACAT
fzd4 -Ex2-F1 HRM fwd	GCATCTAATCCTCTCCTCG
fzd4 -Ex2-R2 HRM rev	CTGGAGAACTGGGAGACAC
fzd4 -Ex2-SP6-F2 Crispr guide	TAGAGAAGGTGCCCATCCCGAT
fzd4 -F2 HRM fwd	GTTCGGGGACGAGGAGGA
fzd4 -Ex1-R2 HRM rev	GTGTGTAGGGTCTGGTTACAGTG
***fzd5* fusion constructs primers**	
fzd5-RFP to *fzd5-DN3-RFP* fwd	GAGATGGCCTCCTCC
fzd5-RFP to *fzd5-DN3-RFP* rev	CAGTGCACAGTTAGGTAG
fzd5-RFP to *fzd5-DN3-myc* fwd	AGCGAAGAAGATCTGTAGAACTATAGTGAGTCGTATTAC
fzd5-RFP to *fzd5-DN3-myc* rev	AATCAGTTTCTGTTCCAGTGCACAGTTAGG
fzd5-RFP to *fzd5-myc* fwd	AGCGAAGAAGATCTGTAGAACTATAGTGAGTCGTATTAC
fzd5-RFP to *fzd5-myc* rev	AATCAGTTTCTGTTCGAGGACATGTGATGAG
**Generation and genotyping of *aamp* U601 allele**	
aamp- exon2 crispr guide	CAACCCAGACCCCGAGCTAGAGG
aamp- exon4 crispr guide	AAGATGACCGAGCGTTCCTGTGG
aamp- exon6 crispr guide	CCCGGCTGCCAGACCACCTGCGG
aamp -exon2-fwd	TAACCCGGATCAATACAGCTG
aamp -exon2-rev	GTGTGTCCTCCAGCTCGATC
***pprc1, cited2, swap70a, swap70b, stm* guides**	
pprc1 -1 exon5 guide	GAGAAAGTCTCTGCATTCGTGGG
pprc1 -2 exon5 guide	AGAGCATGGAGAGCCCATCCTGG
pprc1 -3 exon3 guide	GTTTTCTGGCCACAAGGGTCAGG
cited2 -1 exon2 guide	GCGGGCAGGCAAGTTTCCATTGG
cited2 -2 exon2 guide	AGTCACCAGTTAAACGGGACAGG
cited2 -3 exon2 guide	CGCATGATGGCAATGAACCATGG
swap70a -1 exon2 guide	AAGGACCGGTGTCAACGCAAGGG
swap70a -2 exon2 guide	GGATCTTCAGTATGGTGCACAGG
swap70a -3 exon1 guide	GGGTTTGAGTATTTCTTCCTTGG
swap70b -1 exon1 guide	ACTAAGGGACGAGCTTCTCAAGG
swap70b -2 exon2 guide	TGAAGGTCCGGTTTCCAATCAGG
swap70b -3 exon4 guide	GACCGCCAGACTCTCTCCATGGG
stm-1 exon18 guide	CTTGTCTTCCGAGTCTTTACTGG
stm-2 exon7 guide	AGTGGTGTCAGACGAGTCACCGG
stm-3 exon15 guide	GAATCTGAGTCTGGTCCTTCTGG
***pprc1, cited2, swap70a, swap70b, stm* NGS primers**	
pprc1 -1 exon5 fwd	TCGTCGGCAGCGTCAGATGTGTATAAGAGACAG TGGAGCCCCTTAATAGTTCTCA
pprc1 -1 exon5 rev	GTCTCGTGGGCTCGGAGATGTGTATAAGAGACAG TAGCATCTCCATCTTCCTCCTC
pprc1 -2 exon5 fwd	TCGTCGGCAGCGTCAGATGTGTATAAGAGACAG TAAACACATGCACCCTTACTGC
pprc1 -2 exon5 rev	GTCTCGTGGGCTCGGAGATGTGTATAAGAGACAG ATTTCCTGGAACTCTTTGCTCA
pprc1 -3 exon3 fwd	TCGTCGGCAGCGTCAGATGTGTATAAGAGACAG ATCTTGATGAAGAAAGCGAAGC
pprc1 -3 exon3 rev	GTCTCGTGGGCTCGGAGATGTGTATAAGAGACAG CTAACATGCTTGGACAGGTGAA
cited2 -1 exon2 fwd	TCGTCGGCAGCGTCAGATGTGTATAAGAGACAG GGGAGGGAATATAAACGCAAAT
cited2 -1 exon2 rev	GTCTCGTGGGCTCGGAGATGTGTATAAGAGACAG TAATGGTGATGATGGGAAGGAT
cited2 -2 exon2 fwd	TCGTCGGCAGCGTCAGATGTGTATAAGAGACAG ATCCTTCCCATCATCACCATTA
cited2 -2 exon2 rev	GTCTCGTGGGCTCGGAGATGTGTATAAGAGACAG ACCAGTGACATCAAGACCTCCT
cited2 -3 exon2 fwd	TCGTCGGCAGCGTCAGATGTGTATAAGAGACAG AACATATCTGCACGTCGTGTTT
cited2 -3 exon2 rev	GTCTCGTGGGCTCGGAGATGTGTATAAGAGACAG CATGTGATCTCCCATGATAACG
swap70a -1 exon2 fwd	TCGTCGGCAGCGTCAGATGTGTATAAGAGACAG AACCTGTGCACCATACTGAAGA
swap70a -1 exon2 rev	GTCTCGTGGGCTCGGAGATGTGTATAAGAGACAG ACGCCATTATCACCATCCTAAT
swap70a -2 exon2 fwd	TCGTCGGCAGCGTCAGATGTGTATAAGAGACAG GTGAGTAAATGTTGCATGGCTG
swap70a -2 exon2 rev	GTCTCGTGGGCTCGGAGATGTGTATAAGAGACAG TCGTCATCTTTAAAGTGCTCCTC
swap70a -3 exon1 fwd	TCGTCGGCAGCGTCAGATGTGTATAAGAGACAG CGTGACTCCTGTTTCTGTTGTC
swap70a -3 exon1 rev	GTCTCGTGGGCTCGGAGATGTGTATAAGAGACAG GGGATTTGGATACTTTTCCGTT
swap70b -1 exon1 fwd	TCGTCGGCAGCGTCAGATGTGTATAAGAGACAG TCTGCTAAGGTCATGTGACGAC
swap70b -1 exon1 rev	GTCTCGTGGGCTCGGAGATGTGTATAAGAGACAG GCTGAGATTTTGACACTTTCCC
swap70b -2 exon2 fwd	TCGTCGGCAGCGTCAGATGTGTATAAGAGACAG CTTTCAGGTGCTTTCCCATAAC
swap70b -2 exon2 rev	GTCTCGTGGGCTCGGAGATGTGTATAAGAGACAG TTCAAACCTCAGCATATTTGGA
swap70b -3 exon4 fwd	TCGTCGGCAGCGTCAGATGTGTATAAGAGACAG AGTTCGAACAGTACAAGCTCCA
swap70b -3 exon4 rev	GTCTCGTGGGCTCGGAGATGTGTATAAGAGACAG CTGTTTGAGCACATCCAGAATC
stm-1 exon18 fwd	TCGTCGGCAGCGTCAGATGTGTATAAGAGACAG CGACAAAGACGAAAAACATGAA
stm-1 exon18 rev	GTCTCGTGGGCTCGGAGATGTGTATAAGAGACAG GGTCTTTGGATTCGACAGATTC
stm-2 exon7 fwd	TCGTCGGCAGCGTCAGATGTGTATAAGAGACAG AAAATCCCAAACTGGAACACAC
stm-2 exon7 rev	GTCTCGTGGGCTCGGAGATGTGTATAAGAGACAG CAGCTCGTGTATCAGGTCTTTG
stm-3 exon15 fwd	TCGTCGGCAGCGTCAGATGTGTATAAGAGACAG CAAAATAATGATAACGACGGATGA
stm-3 exon15 rev	GTCTCGTGGGCTCGGAGATGTGTATAAGAGACAG CTGCCTGATTGATGATGTTGTT

Disease Models & Mechanisms • Supplementary information
